# Effect of Delivery Tube Diameter on Melt Breakup and Powder Refinement During Water Atomization of FeSiCr Alloy Powder

**DOI:** 10.3390/ma19163455

**Published:** 2026-08-14

**Authors:** Yifan Li, Pu Wang, Jiaquan Zhang

**Affiliations:** 1 School of Metallurgy and Ecological Engineering, University of Science and Technology Beijing, Beijing 100083, China; 2 School of Materials and Physics Science, Nanchang University, Nanchang 330031, China

**Keywords:** water atomization, delivery tube diameter, FeSiCr alloy powder, industrial validation, powder refinement

## Abstract

**Highlights:**

- The 4 mm delivery tube balances powder refinement, size uniformity, and stable continuous production.
- As tube diameter increases, melt momentum rises, primary breakup shifts downstream, and particle size increases markedly.
- The coupled VOF-DPM model accurately captures breakup and droplet evolution, as validated by industrial trials.

**Abstract:**

Water atomization is widely used for producing FeSiCr alloy powder, in which melt delivery conditions strongly influence jet breakup and powder refinement. In this study, a coupled volume of fluid-discrete phase model (VOF-DPM) was established to investigate the effect of delivery tube diameter (3, 4, 5, and 6 mm) on flow characteristics, interfacial instability, and droplet evolution at a constant water pressure of 120 MPa, and the simulation results were validated by industrial trials. As the tube diameter increased from 3 to 6 mm, the melt mass flow rate rose from 0.100 to 0.368 kg/s, the primary breakup position shifted downstream from 94.6 to 236.5 mm, and the peak negative pressure along the centerline decreased from −41.12 to −31.65 kPa. The simulated average particle size increased from 9.176 to 22.791 μm, while the experimentally measured mean particle size increased from 9.0 to 19.6 μm and the fine-powder yield decreased from 43.77% to 22.28%. Although the 3 mm tube produced the finest powder, it showed a higher tendency for clogging and unstable melt delivery. Overall, the 4 mm delivery tube provided the best balance between powder refinement, size uniformity, and production stability.

## 1. Introduction

Soft magnetic composites (SMCs) are advanced functional materials fabricated by powder metallurgy, typically involving powder insulation treatment, compaction, and subsequent heat treatment [1]. Compared with conventional silicon steels and ferrites, SMCs offer distinct advantages for high-frequency and lightweight power electronic devices because of their three-dimensional magnetic flux paths, compact geometry, and good machinability [2,3,4,5]. With the continued increase in operating frequency and the ongoing miniaturization of electronic devices, stricter requirements have been imposed on the low-loss performance of soft magnetic materials under high-frequency conditions [6,7]. In this context, powder characteristics, particularly particle size distribution, play a decisive role in densification behavior and magnetic domain configuration, thereby directly influencing the overall performance of SMCs. Therefore, increasing the yield of fine powder has become an important route for improving the performance of soft magnetic composites [8,9,10].

In addition, the powder preparation process has a significant influence on particle size distribution, particle morphology, and internal microstructure. Among the available methods, water atomization has become one of the most important techniques for producing iron-based soft magnetic powders because of its high production efficiency, low cost, and suitability for large-scale industrial manufacturing [11]. In this process, high-pressure water jets interact with the molten metal stream, causing the liquid column to disintegrate rapidly into fine droplets under the combined effects of impact and shear; these droplets then cool and solidify during flight to form metal powders. In relation to this process, Liu et al. [12] prepared Fe-based amorphous magnetic powders by water atomization and analyzed the relationship between melt jet breakup behavior and powder particle size, showing that high-velocity water jets can markedly enhance melt disintegration and increase the proportion of fine powder. Subsequently, Li et al. [13] proposed a combined spinning-water atomization process for the preparation of Fe-Si-B-P-Cu amorphous powders, with the aim of improving glass-forming ability and optimizing particle size distribution. Their results indicated that this method could produce powders with a more uniform particle size distribution and favorable soft magnetic properties. These studies demonstrate that the breakup behavior of the melt column during water atomization directly affects the resulting powder size characteristics, while the hydrodynamic conditions in the atomization zone play a critical role in achieving powder refinement.

At the level of dynamic mechanisms, the breakup evolution of a molten metal column under the impact of high-velocity water jets is governed by the combined effects of momentum matching at the water-melt interface, fluid instabilities such as Kelvin-Helmholtz instability, and the local topology of the pressure field [14]. Dhokey et al. [15] investigated the effects of water pressure and nozzle apex angle on the particle size of water-atomized copper powder through experiments and theoretical modeling, and established a predictive relationship between processing parameters and particle size. Their results showed that increasing water pressure and optimizing the spray angle could significantly reduce the mean particle size. Asgarian et al. [16] examined the influence of water spray configuration on atomization behavior and found that a proper nozzle arrangement could enhance interfacial disturbance and promote melt jet breakup. In addition, Han et al. [17], in their study of the flow field characteristics of atomizing nozzles, reported that variations in water supply pressure altered the local flow structure and shear intensity, thereby affecting the particle refinement capability. These studies indicate that optimizing the flow field structure and geometric parameters of the atomization system can effectively improve melt breakup efficiency and refine the powder particle size distribution. 

In practical industrial production, in addition to processing parameters such as water pressure, nozzle structure, and melt temperature, the configuration of the melt delivery system also has an important influence on atomization performance. As the key component connecting the melting stage and the atomization chamber, the diameter of the delivery tube directly determines the initial mass flow rate and momentum flux of the melt entering the atomization zone, thereby fundamentally governing the momentum exchange behavior between the water jets and the molten metal during the primary breakup stage [18,19]. When the momentum of the melt jet is excessively high, its penetration capability increases, while the enveloping and shearing effects of the water jets are weakened, resulting in reduced breakup efficiency; conversely, an excessively low melt flow rate may impair the stability of melt delivery. With the development of computational fluid dynamics (CFD), numerical simulation has become an important tool for investigating the mechanisms of metal atomization. Zhou et al. [20] used numerical simulation to study the breakup, cooling, and solidification of a metal jet in water, revealing the influence of flow field characteristics on droplet formation. Wang et al. [21] further pointed out that changes in nozzle geometric parameters could significantly affect jet velocity distribution and flow field stability. However, compared with studies focusing on nozzle structure and water pressure, systematic investigations on melt delivery system parameters, particularly the effect of delivery tube diameter on breakup behavior during water atomization, remain relatively limited.

Based on the above research background, the present study focuses on the water atomization process for FeSiCr soft magnetic alloy powder, with particular emphasis on the influence of delivery tube diameter as a key structural parameter on atomization behavior and powder size characteristics. By constructing a three-dimensional multiphase flow model of the air-water-melt system and employing a coupled VOF-DPM approach, the flow field characteristics, interfacial breakup features, and droplet size evolution under different delivery tube diameters were systematically simulated and analyzed. The simulation results were further validated through industrial water atomization experiments. Through this combined numerical and experimental investigation, the mechanism by which delivery tube diameter regulates momentum matching between the water jets and molten metal, as well as interfacial instability behavior, was clarified, thereby providing a theoretical basis for equipment structure optimization and process control in the production of high-quality FeSiCr soft magnetic powders.

## 2. Models and Methods

**Figure 1:**
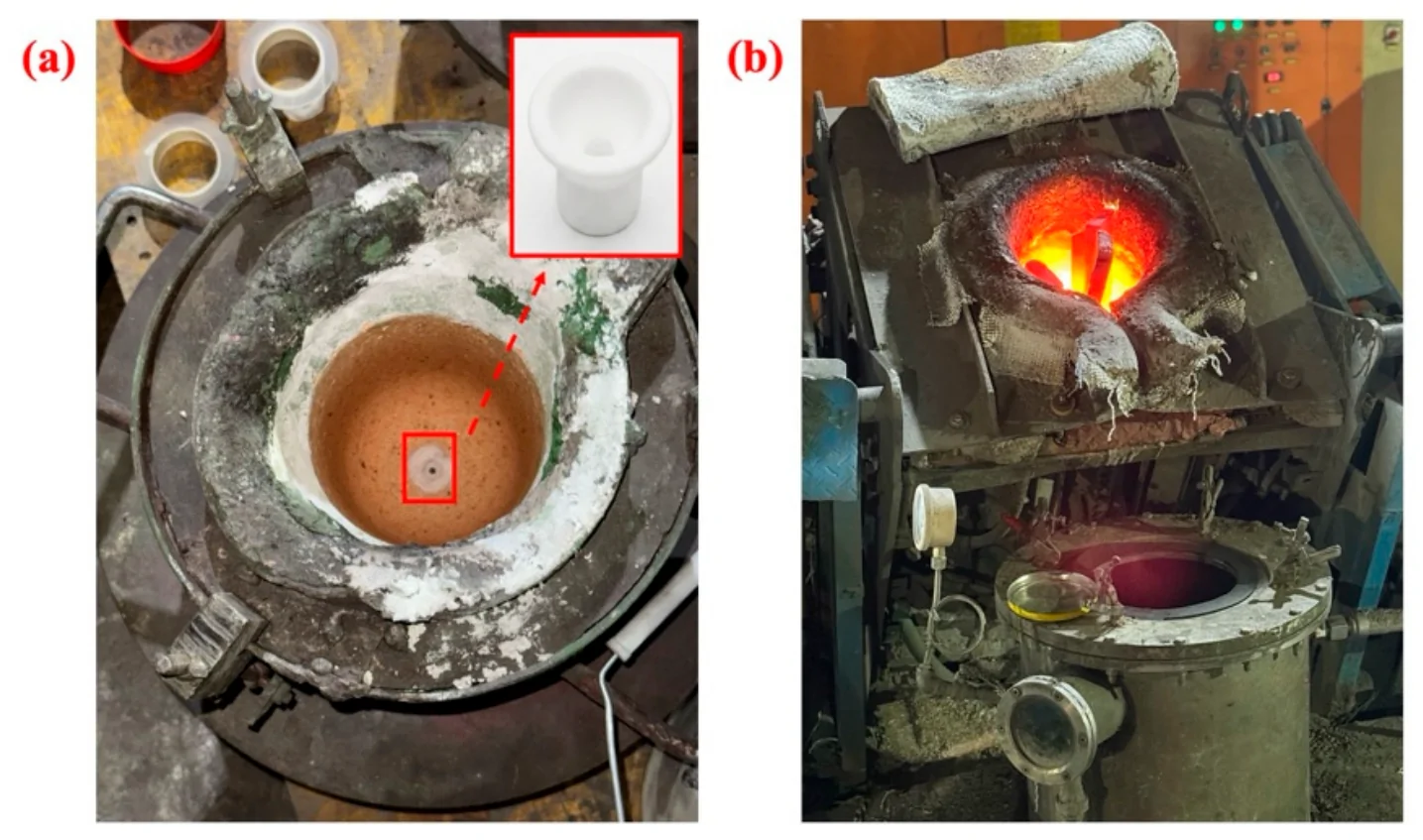
Photographs of the industrial water-atomization equipment: (**a**) actual installation position of the melt delivery tube in the tundish; and (**b**) melting and pouring operation prior to water atomization.

In the industrial production of FeSiCr powder by water atomization, the equipment mainly consists of a melting furnace, a tundish, a melt delivery tube, and an atomization chamber. The nominal chemical composition of the FeSiCr alloy used in this study was 89.0 wt.% Fe, 5.5 wt.% Si, and 5.5 wt.% Cr. Figure 1a shows the actual installation position of the delivery tube at the center of the tundish bottom, while Figure 1b shows the melting and pouring operation before atomization. During production, the FeSiCr alloy is first melted in the furnace and heated to the target pouring temperature. The molten alloy is then slowly poured into the tundish and flows downward through the delivery tube into the atomization chamber. After leaving the delivery tube, the continuous melt stream interacts with the high-pressure water jets and is broken into droplets, which subsequently cool and solidify to form alloy powder. Therefore, the delivery tube acts as the connecting component between the melting stage and the atomization stage and controls the initial melt flow rate and velocity entering the atomization chamber.

**Figure 2:**
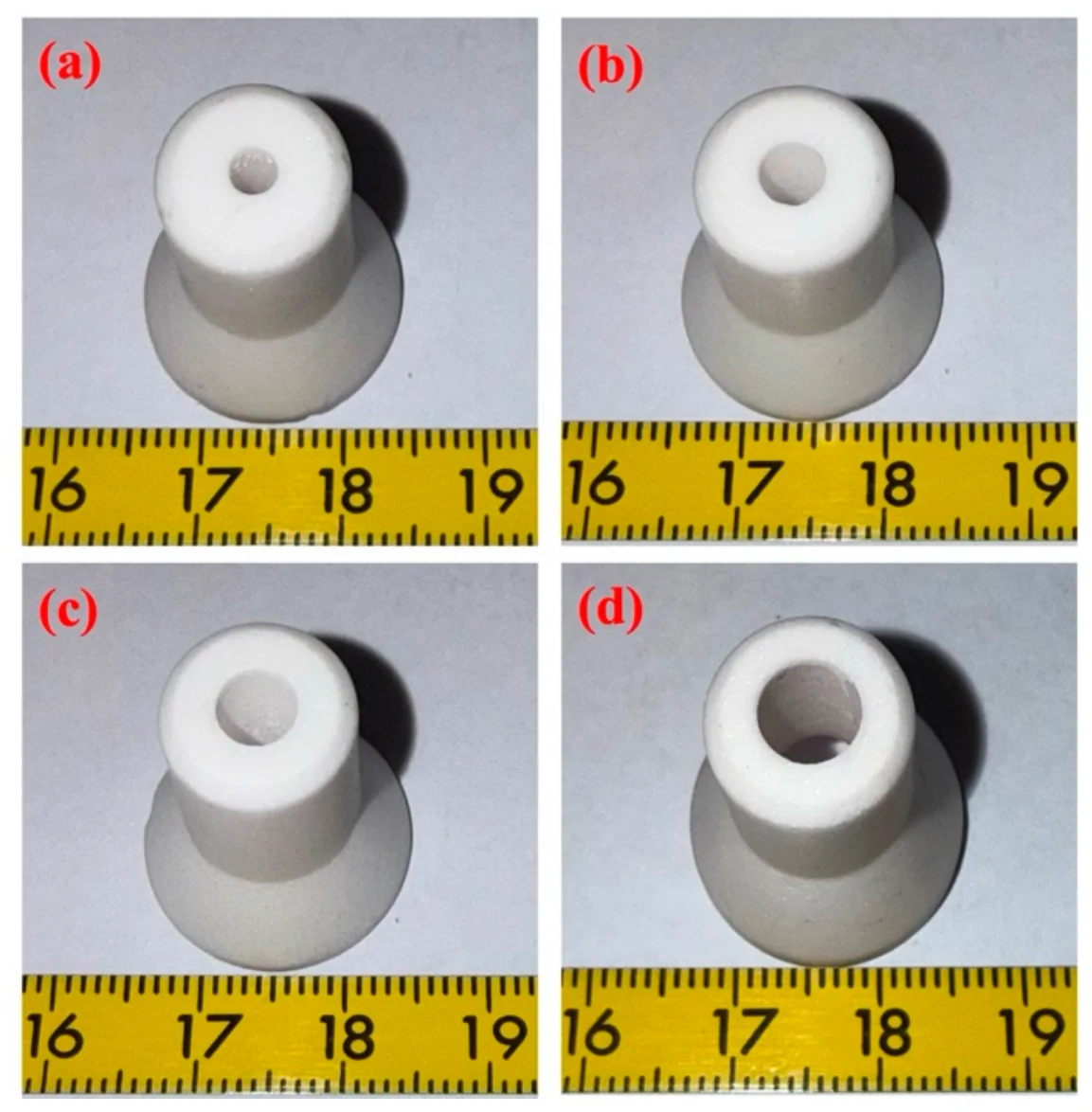
Photographs of the zirconia melt delivery tubes with different inner diameters: (**a**) 3 mm; (**b**) 4 mm; (**c**) 5 mm; and (**d**) 6 mm.

In this study, three-dimensional geometric models were established for the delivery tubes commonly used in industrial production, with diameters of 3, 4, 5, and 6 mm, as shown in Figure 2. In actual production, the melt flow rate increases to different extents with increasing delivery tube diameter. Under the condition of a constant melt level in the tundish, repeated production results were used to summarize the relationship between melt flow rate and delivery tube diameter, as presented in Table 1.

The outlet of the high-pressure water nozzle used in this study was flat. The same nozzle outlet geometry was used for all investigated conditions; therefore, its effect was kept constant, allowing the influence of delivery tube diameter to be isolated. All delivery tubes used in the industrial experiments were made of zirconia of the same material grade. The external geometry, total length, and installation conditions of the tubes were kept identical, and only the inner diameter was varied among 3, 4, 5, and 6 mm. Therefore, the delivery-tube material was treated as a controlled parameter and was not investigated as an independent variable.

**Figure 3:**
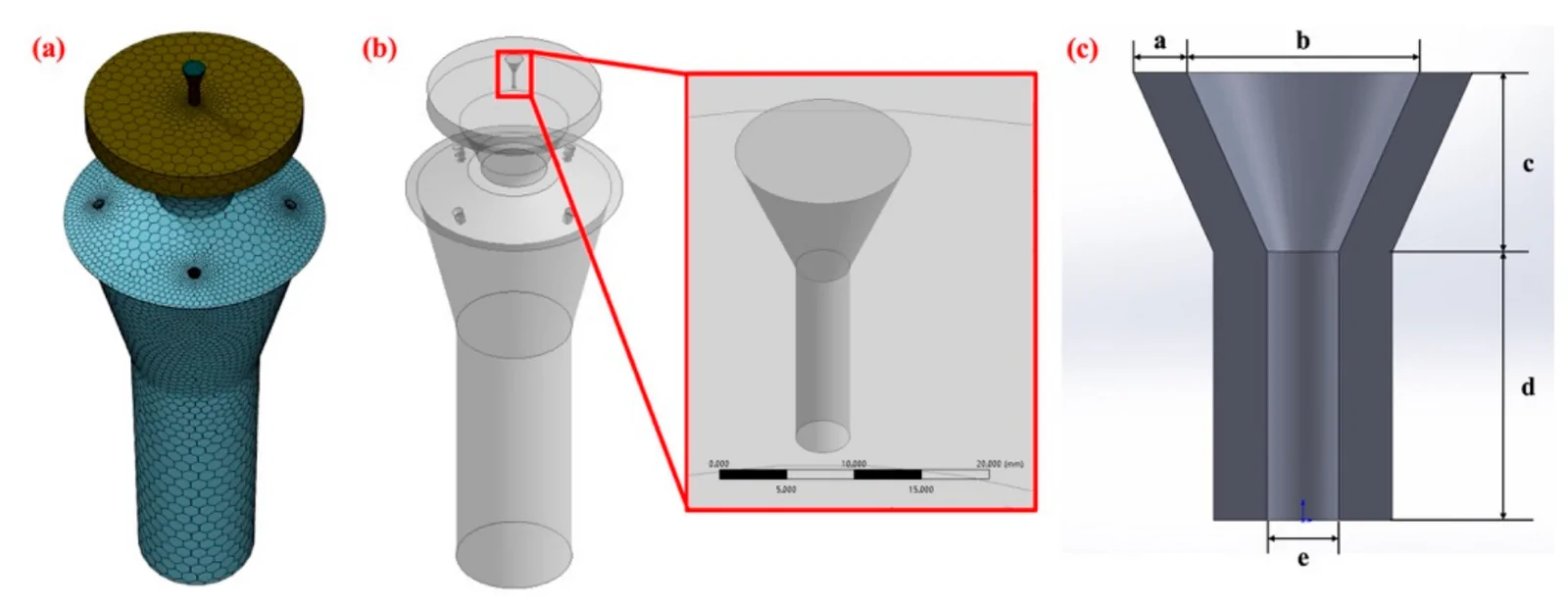
(**a**) Computational mesh; (**b**) geometric model; (**c**) engineering drawing of the delivery tube.

Figure 3 shows the simplified model and the corresponding computational mesh. The industrial atomization system employs four identical flat water nozzles, which are uniformly arranged around the melt-stream centerline at 90° intervals. This four-nozzle configuration is the fixed structural design of the existing industrial equipment and was not selected or optimized in the present study. The circumferentially symmetric arrangement provides approximately balanced radial momentum input to the molten-metal stream and reduces its lateral deflection during atomization. To isolate the effect of delivery-tube diameter, the number, outlet geometry, installation angle, and water-supply conditions of the nozzles were kept unchanged for all investigated cases. In actual production, water enters the spray distributor from a single direction and is subsequently distributed to the four nozzles. Although slight flow-rate differences may arise because of the different distances between the distributor inlet and individual nozzles, these differences were neglected under the high-pressure operating condition. Therefore, the four nozzles were assigned identical inlet conditions and were assumed to discharge simultaneously in the numerical model.

To ensure consistency in the comparison among different conditions, all geometric parameters were kept constant except for the inner diameter of the delivery tube, including the tube length, nozzle installation height, spray angle, distance from the nozzle to the centerline, and axial distance from the bottom of the tundish to the nozzle position. The corresponding geometric parameters are listed in Table 2. The parameters a–e in Table 2 correspond directly to the dimension labels in Figure 3c. Parameters a, b, c, and d denote the upper rim width, total inlet width, tapered-section height, and straight-section length of the delivery tube, respectively, while e denotes the inner diameter of the delivery tube. All dimensions except e were kept constant among the investigated cases.

During mesh generation, the Ansys Fluent Meshing 2023 R1 module was employed, and local mesh refinement was applied at the outlet of the delivery tube and the outlets of the high-pressure flat water nozzles in the atomization chamber. The mesh size in the refined regions was set to 0.08 mm. For the boundary conditions used in the simulation, the inlet of each high-pressure water nozzle was specified as a mass-flow inlet with a flow rate of 0.383 kg/s, corresponding to the industrial operating condition of 120 MPa water pressure. The four nozzles were assigned identical inlet parameters and were assumed to operate synchronously and symmetrically. This idealized boundary condition represents the nominal operating state of the industrial four-nozzle atomization unit and prevents nozzle-to-nozzle flow imbalance from being introduced as an additional variable in the analysis of the main flow field. The inlet of the delivery tube was also defined as a mass-flow inlet, and the melt flow rates corresponding to different delivery tube diameters were taken from the industrial statistical data (see Table 1). The bottom of the model was set as a pressure outlet, while all other walls were treated as adiabatic no-slip boundaries.

**Figure 4:**
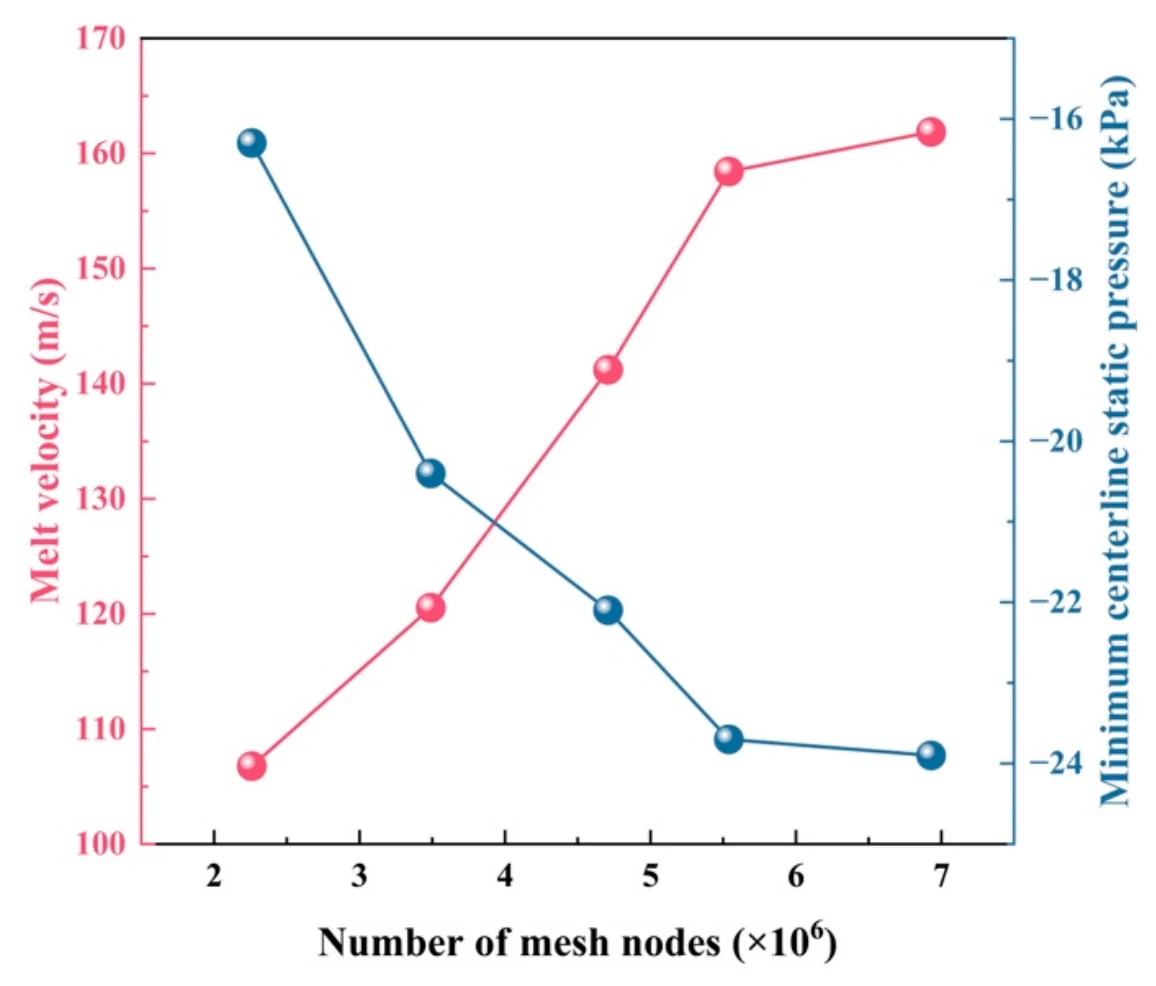
Grid-independence test based on the melt velocity at the primary breakup position and the minimum centerline static pressure.

The grid independence test is a critical step in ensuring the reliability of numerical simulation results. In this study, meshes with 2.26 × 10^6^, 3.49 × 10^6^, 4.71 × 10^6^, 5.54 × 10^6^, and 6.93 × 10^6^ nodes were examined. All data presented in Figure 4 were obtained from the numerical calculations performed in this study rather than from the literature. The melt velocity at the primary breakup position and the minimum static pressure along the model centerline were selected as the monitored quantities. As the number of mesh nodes increased, the variations in the velocity at the melt breakup point and the minimum static pressure along the model centerline gradually diminished. Considering both accuracy and computational efficiency, the mesh with 5.54 × 10^6^ nodes was selected for the subsequent simulations, as it ensured adequate numerical accuracy while reducing computational cost and time.

The simulations in this study were performed using the finite-volume-based CFD software Ansys Fluent 2022 R1. To simplify the actual atomization process while maintaining computational accuracy and reducing computational cost and time, the following assumptions were made:

(1) all walls were treated as smooth adiabatic no-slip boundaries;

(2) the velocity and pressure of the water jets were assumed to remain constant during atomization;

(3) the density, dynamic viscosity, and interfacial tension of the FeSiCr melt were treated as temperature-independent constants evaluated at the industrial pouring temperature of 1953.15 K. The adopted melt density and dynamic viscosity were 7080 kg·m^−3^ and 5.76 × 10^−3^ Pa·s, respectively, while the air–FeSiCr and water–FeSiCr interfacial tension coefficients were 1.086 and 1.524 N·m^−1^, respectively;

(4) phase change and evaporation effects between different phases were neglected in the VOF model;

(5) in the DPM model, melt droplets were assumed to be spherical, and droplet-droplet collision and coalescence were neglected, with only drag force, gravity, and the turbulent force induced by the water flow being considered.

The governing equations related to the atomization model are summarized in Table 3 [22], and detailed descriptions of these equations can be found in our previous studies [23,24,25,26].

The thermophysical properties of air, water, and FeSiCr melt used in this study are listed in Table 4. The thermophysical properties of the FeSiCr alloy were obtained through a combination of thermodynamic calculations and experimental measurements. The calculations were performed using thermodynamic software such as JMatPro 7.0 based on the nominal alloy composition of 89.0 wt.% Fe, 5.5 wt.% Si, and 5.5 wt.% Cr, and the calculated values were supplemented and verified using available experimental measurements. The properties of air and water were obtained from the standard material database incorporated in the numerical software (Ansys Fluent 2023 R1) and were consistent with commonly accepted experimental data. The thermophysical properties of air, water, and the FeSiCr melt used in the numerical model are listed in Table 4. For the FeSiCr melt, the density and dynamic viscosity were set to 7080 kg·m^−3^ and 5.76 × 10^−3^ Pa·s, respectively. The interfacial tension coefficients for the air–water, air–FeSiCr, and water–FeSiCr interfaces were 0.072, 1.086, and 1.524 N·m^−1^, respectively. These values were treated as temperature-independent constants evaluated at the industrial pouring temperature of 1953.15 K and were applied throughout the 10 ms transient atomization calculation. Because the present study focuses on the effect of delivery-tube diameter on flow-field characteristics and melt-breakup behavior over a short time scale, the temperature dependence of the thermophysical properties was neglected as a modeling simplification.

For the pure water flow field, the multiphase flow was solved using a steady-state VOF model, while turbulence was described by the realizable k-ε model. The coupled algorithm was employed, and first-order upwind discretization was applied to the momentum, turbulent kinetic energy, and dissipation rate equations. During the continuous-phase solution stage, convergence of the steady pure water flow field was determined by the simultaneous stabilization of the residuals of all governing equations and the flow parameters at key monitoring locations. After the water flow field reached a steady state, the melt phase was introduced for transient coupled calculations to simulate the interfacial breakup process after the molten metal entered the atomization zone.

**Figure 5:**
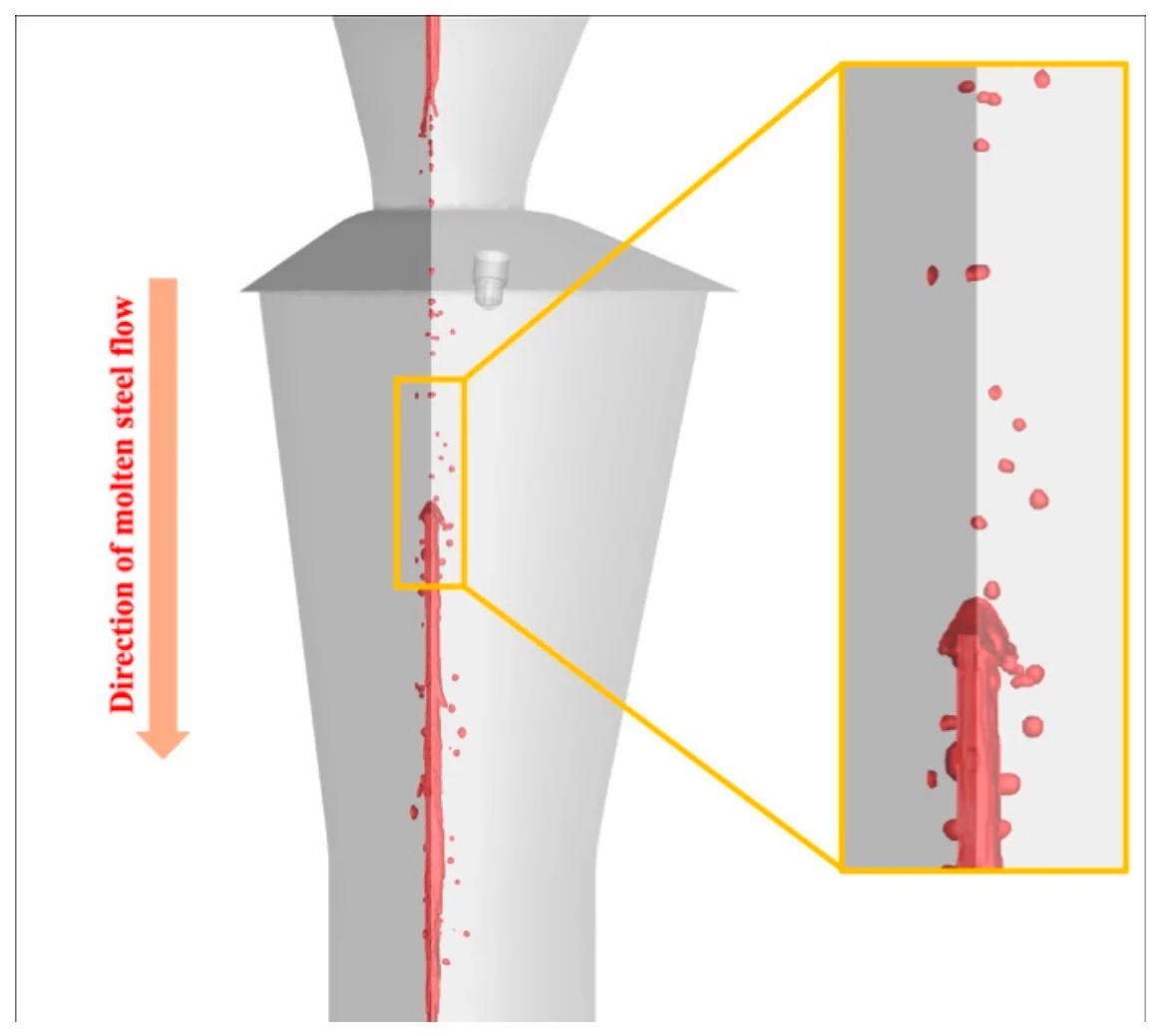
Instantaneous VOF result illustrating the definition of the primary breakup position of the molten-metal column.

To ensure consistency in the comparison among different operating conditions, the primary breakup position was defined as the first location, along the downward melt-flow direction, where the continuous molten-metal column changed into a clearly discontinuous state consisting of separated liquid segments or droplets. Figure 5 presents an instantaneous phase-distribution result obtained from the transient VOF calculation. The apparent asymmetry of the molten-metal column is caused by instantaneous turbulent fluctuations, local pressure disturbances, and interfacial instability under the action of the high-velocity water flow. It does not indicate any geometric asymmetry in the delivery tube, nozzle arrangement, or computational domain. The arrow in Figure 5 indicates the downward melt-flow direction, and the enlarged inset shows this transition from the continuous liquid column to the first clearly discontinuous breakup region. The axial distance from this point to the bottom of the tundish was used as the characteristic parameter, and the corresponding values are listed in Table 5. In the DPM model, the particle injection location was determined based on the initial primary breakup region obtained from the VOF simulation. The initial particle temperature was set to the melt pouring temperature of 1953.15 K, and the initial particle velocity was matched to the local continuous-phase flow condition. Secondary droplet breakup was treated using the Wave model to capture the further breakup and refinement of droplets under the action of the high-velocity water flow. During the transient calculation stage, an adaptive time-stepping method was adopted, with a minimum time step of 1 × 10^−7^ s and a total simulation time of 10 ms.

## 3. Simulation Result

To systematically clarify the effect of delivery tube diameter on the breakup behavior during water atomization of FeSiCr alloy, numerical simulations were carried out for different delivery tube diameters while keeping the nozzle structure, spray angle, and water supply conditions unchanged. The melt mass flow rate for each condition was taken from the industrial statistical data listed in Table 1. Since the water-side input remained constant, changing the delivery tube diameter essentially altered the mass flow rate and initial momentum flux of the melt jet, thereby regulating the momentum matching between water and molten metal in the atomization zone and further affecting the primary breakup behavior of the liquid column.

**Figure 6:**
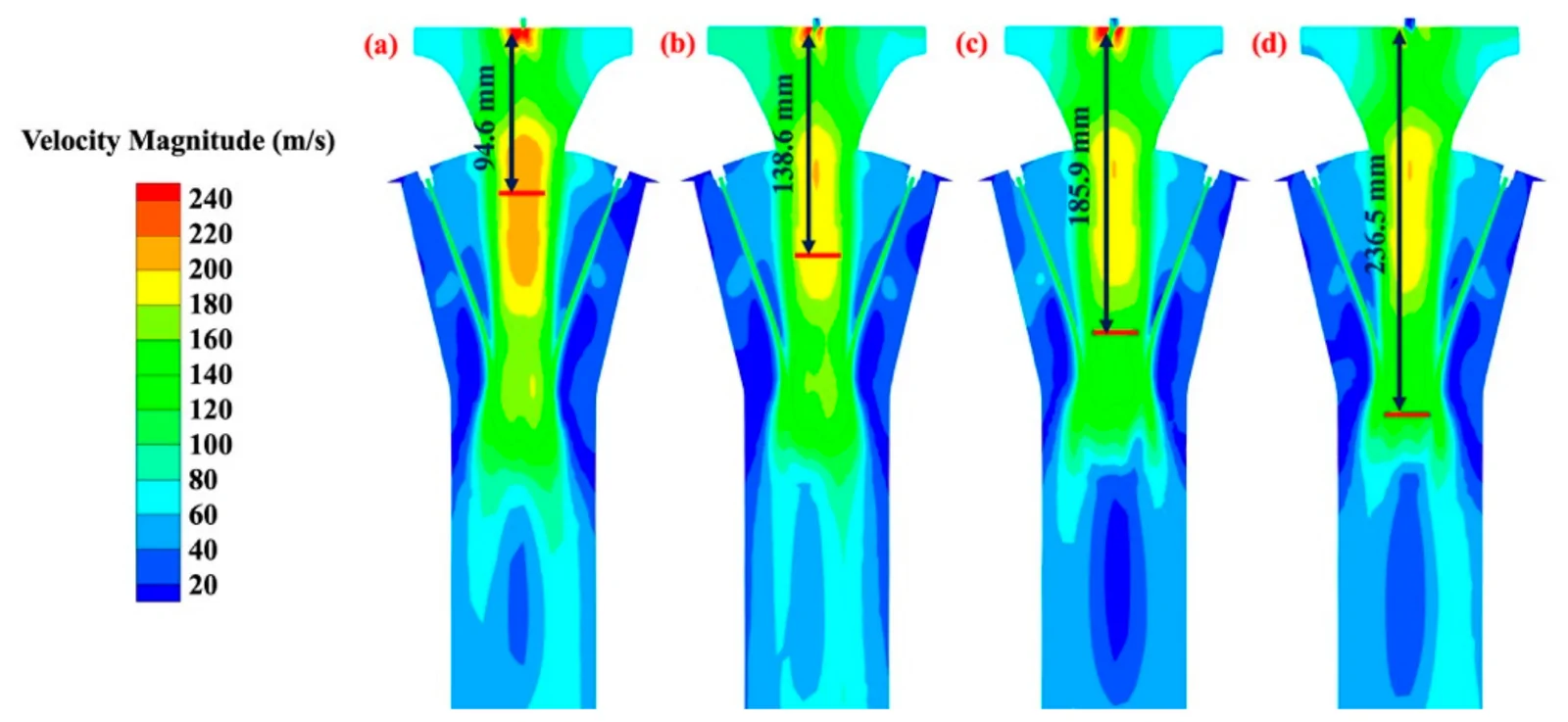
Velocity contour on the model’s axial plane: (**a**) 3 mm; (**b**) 4 mm; (**c**) 5 mm; (**d**) 6 mm.

Table 5 and Figure 6 present the distance from the primary breakup point of the molten metal stream to the bottom of the tundish, together with the velocity contours on the central plane of the numerical model under different delivery tube diameters. The corresponding primary breakup position in each velocity contour is indicated by a line and an arrow. It can be seen that as the delivery tube diameter increased from 3 to 6 mm, the primary breakup position continuously shifted downstream, moving from 94.6 to 236.5 mm. This result indicates that increasing the delivery tube diameter enhanced the inertia of the melt jet and significantly increased the axial penetration capability of the liquid column, while the constraining and breakup-promoting effects of the water jets in the near-nozzle region gradually weakened. When the delivery tube diameter was 3 mm, the melt flow rate was the lowest, only 0.1 kg/s. Under this condition, the high-velocity water jets formed a dominant flow field in the axial region, and a pronounced negative-pressure entrainment zone developed at the jet impingement region. As a result, the molten metal was subjected to strong stretching and disturbance over a relatively short distance, and the primary breakup position was located above the high-pressure water nozzles. This indicates that under the small-diameter delivery tube condition, the water flow exerted strong enveloping, entraining, and shearing effects on the molten metal column, thereby promoting earlier instability and breakup of the liquid column.

As the delivery tube diameter increased to 4 and 5 mm, the melt flow rate increased, and the liquid column was still able to maintain strong interaction with the high-velocity water jets while sustaining continuous melt delivery. In particular, under the 4 mm condition, a relatively well-coordinated balance was achieved between the penetration capability of the liquid column and the shearing capability of the water flow. Although the primary breakup position shifted downstream compared with that under the 3 mm condition, it still remained within a favorable breakup region. In contrast, under the 6 mm condition, the liquid column retained a relatively intact columnar morphology over a longer distance, indicating that the high-momentum melt significantly weakened the breakup-promoting effect of the water flow in the near-field region, resulting in a markedly delayed primary breakup.

**Figure 7:**
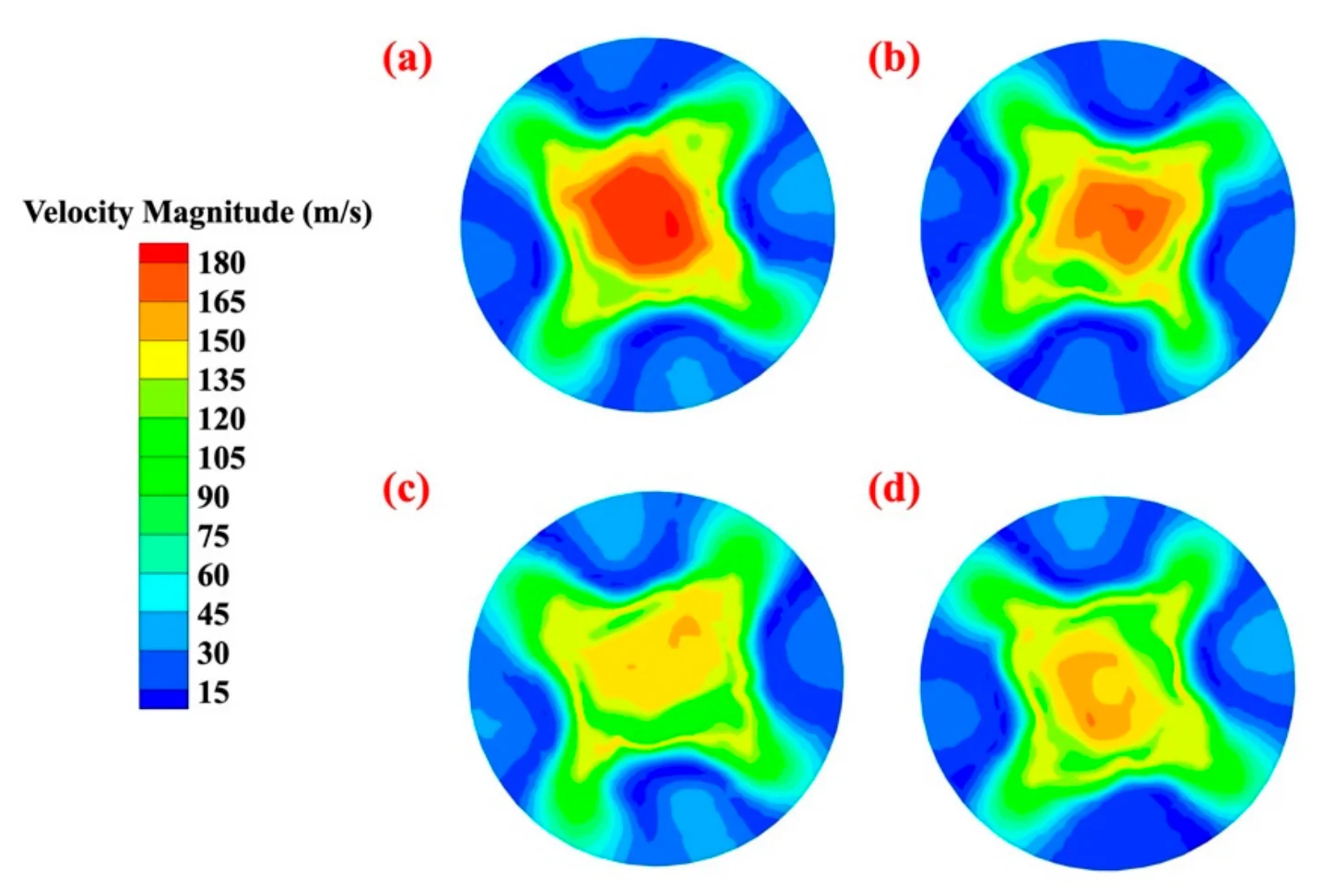
Velocity contours at the breakup section of the model: (**a**) 3 mm; (**b**) 4 mm; (**c**) 5 mm; and (**d**) 6 mm.

Figure 7 shows the velocity contours in the breakup region. It can be observed that under the 3 and 4 mm conditions, a distinct high-velocity water “enveloping” structure was formed in the breakup zone, generating extremely large local velocity gradients and strong shear bands, which caused the molten metal to rapidly disintegrate into fine droplets. Under the 5 mm condition, the shear zone became broader, but the local maximum velocity gradient decreased, indicating a reduction in the secondary breakup capability. Under the 6 mm large-diameter condition, the breakup cross section exhibited large unbroken melt agglomerates together with a highly non-uniform velocity distribution. This phenomenon indicates that the droplet refinement mechanism had shifted from continuous interfacial shear-induced instability to localized random impact-induced fragmentation, leading to a substantial decrease in breakup efficiency.

**Figure 8:**
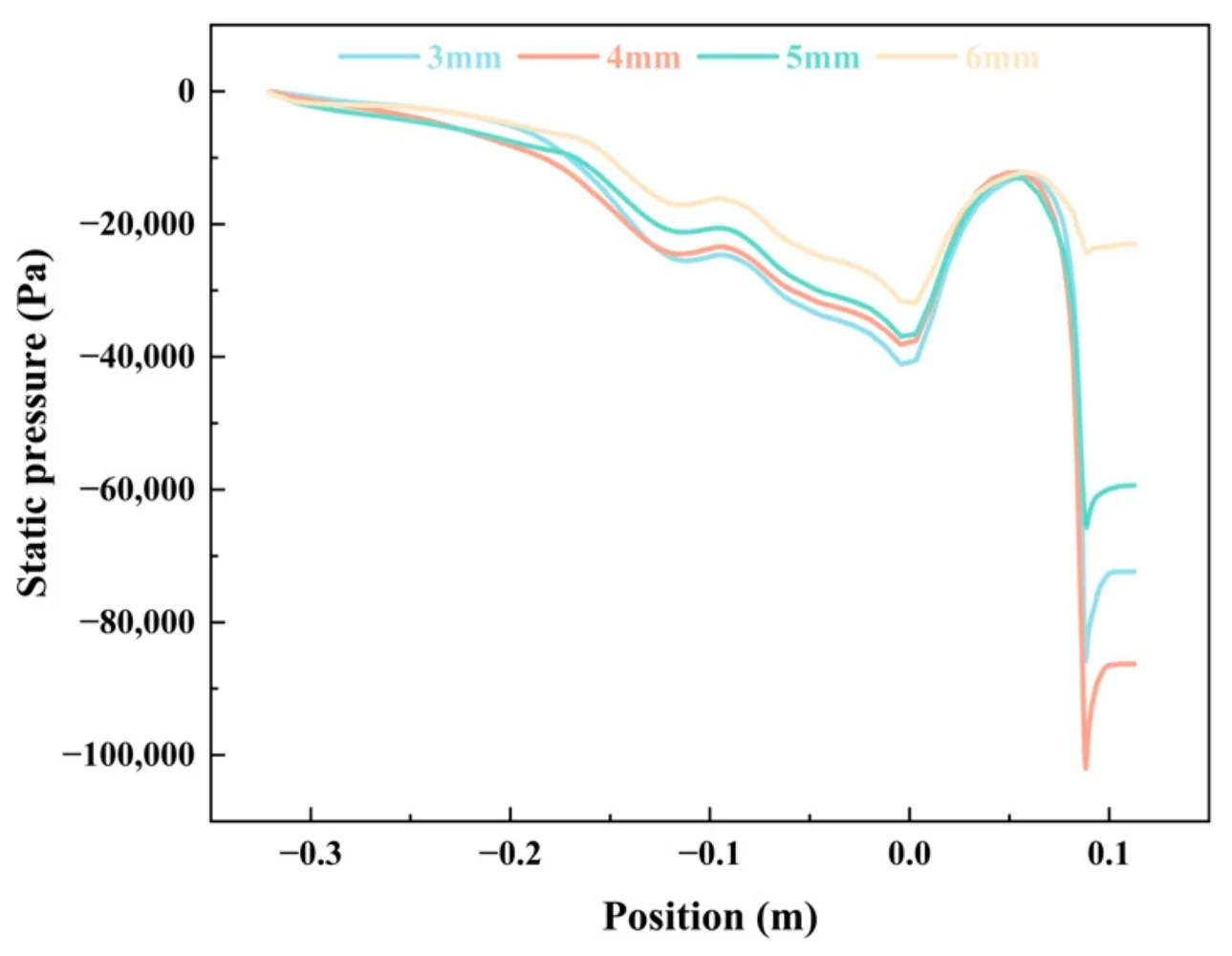
Static pressure distribution along the model centerline.

**Figure 9:**
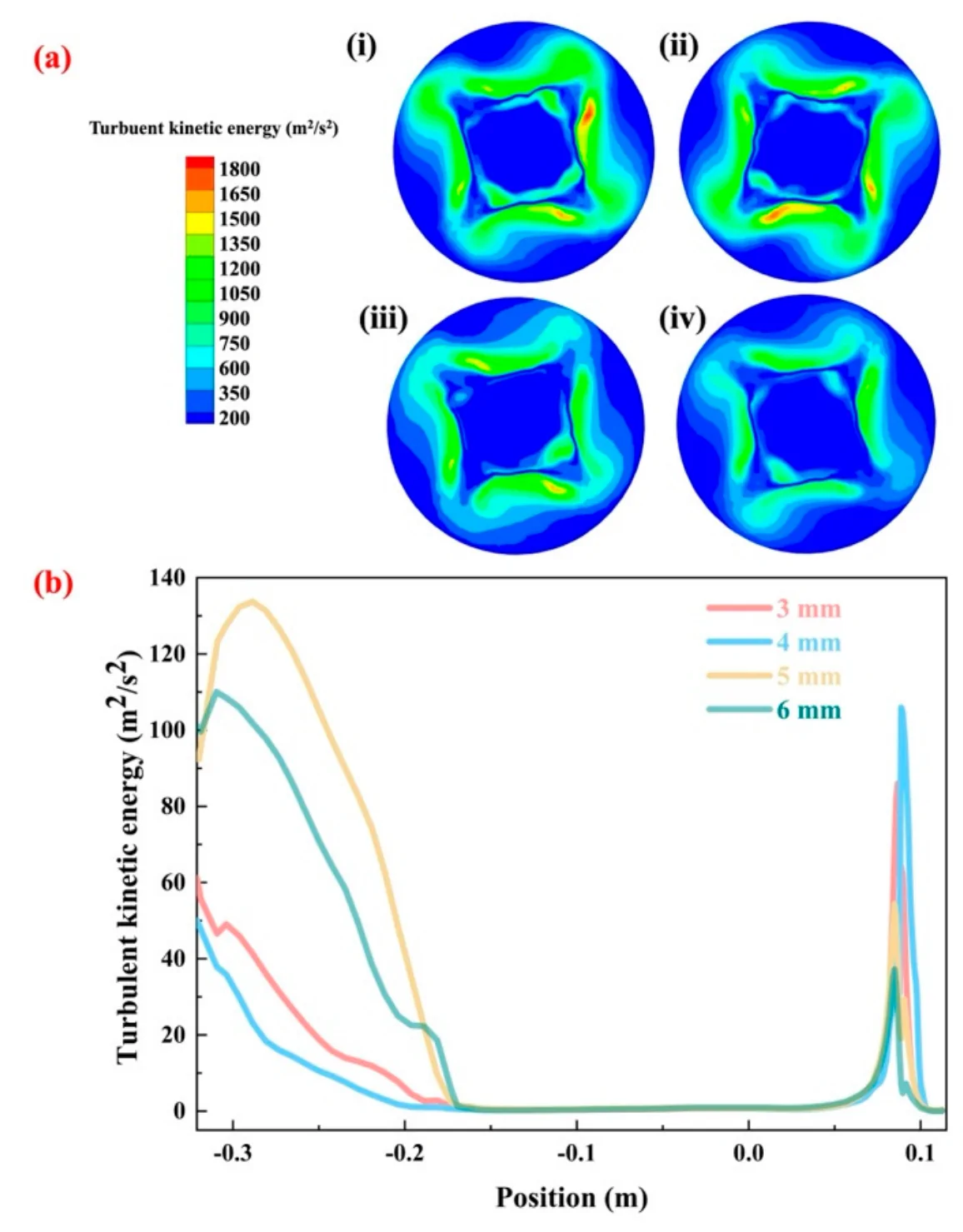
(**a**) Turbulent kinetic energy contours at the breakup section of the model: (i) 3 mm; (ii) 4 mm; (iii) 5 mm; and (iv) 6 mm; (**b**) turbulent kinetic energy distribution along the model centerline.

For clarity, the model centerline was defined as the vertical symmetry line passing through the center of the delivery tube and extending axially through the atomization chamber. The axial coordinate along this line was measured from the bottom of the tundish, which was defined as z = 0. The downward direction into the atomization chamber was defined as positive, whereas the upward direction was defined as negative. The term “Position” in Figure 8 and Figure 9 refers to this axial coordinate.

Figure 8 presents the static pressure distribution along the model centerline. The results show that as the delivery tube diameter increased, the peak negative pressure along the centerline gradually decreased, and the pressure recovery position shifted downstream. Under the 3 and 4 mm conditions, the peak negative pressures were −41.12 and −38.13 kPa, respectively. A pronounced negative-pressure zone was formed in the region where the water jets converged, which enhanced the entrainment and stretching effects on the molten metal, thereby accelerating interfacial instability and liquid column breakup. In contrast, under the 6 mm condition, the magnitude of the negative pressure was significantly reduced to −31.65 kPa, indicating that the breakup of the molten metal relied more on direct impact and inertial effects, while the development of interfacial wave disturbances was constrained. This evolution of the pressure field is highly consistent with the velocity field analysis, further confirming the decisive role of momentum matching in atomization efficiency.

To further elucidate the intrinsic reasons for the differences in liquid column instability and droplet refinement capability under different delivery tube diameters, Figure 9 presents the turbulent kinetic energy contours at the breakup section and the turbulent kinetic energy distribution curves along the model centerline. As shown in Figure 9a, the turbulent kinetic energy distribution in the breakup region varied significantly with delivery tube diameter. Under the 3 mm condition, the maximum turbulent kinetic energy at the breakup section reached 1761.02 m^2^/s^2^, and the high-turbulent-kinetic-energy region was mainly concentrated near the interface where the water flow intersected with the liquid column, indicating that local disturbances were the most intense under this condition and that the impact and shear effects of the water flow on the liquid column were the strongest. Under the 4 mm condition, the maximum turbulent kinetic energy was 1660.18 m^2^/s^2^. Although this value was slightly lower than that under the 3 mm condition, the high-turbulent-kinetic-energy region was distributed more continuously and uniformly, suggesting that this condition could provide a more stable breakup environment while still maintaining strong disturbance intensity. In contrast, under the 5 and 6 mm conditions, the maximum turbulent kinetic energy decreased to 1542.17 and 1452.82 m^2^/s^2^, respectively, representing reductions of 7.11% and 12.49% relative to the 4 mm condition. These results indicate that as the delivery tube diameter increased, the local disturbances induced by the water flow in the breakup region were progressively weakened.

Further insight can be obtained from the turbulent kinetic energy distribution along the model centerline shown in Figure 9b. The peak centerline turbulent kinetic energy values under the 3, 4, 5, and 6 mm conditions were 84.74, 105.51, 54.34, and 37.19 m^2^/s^2^, respectively, and the corresponding peak positions were located 25.71, 23.79, 27.63, and 30.51 mm below the bottom of the tundish. Unlike the maximum turbulent kinetic energy at the breakup section, the peak centerline turbulent kinetic energy reached its highest value under the 4 mm condition, representing an increase of approximately 24.51% compared with the 3 mm condition and increases of about 94.16% and 183.70% compared with the 5 and 6 mm conditions, respectively. This result indicates that, under the 4 mm condition, a stronger and more concentrated core disturbance region could be formed near the centerline of the atomization zone, which was more favorable for stable and effective instability development and breakup of the liquid column in the near-field region.

It can also be observed that as the delivery tube diameter further increased from 4 to 5 and 6 mm, the peak centerline turbulent kinetic energy not only decreased markedly, but its peak position also shifted downstream from 23.79 to 27.63 and 30.51 mm. This suggests that as the melt momentum continued to increase, the ability of the water flow to effectively disturb the liquid column in the near-field region was weakened, and the main disturbance zone migrated progressively downstream, making it more difficult for sufficiently strong interfacial instability to develop near the nozzles. This trend is consistent with the previously observed downstream shift in the primary breakup position from 94.6 to 236.5 mm and the decrease in the peak negative pressure along the centerline from −41.12 to −31.65 kPa, further indicating that the near-field breakup capability of the liquid column progressively deteriorated with increasing delivery tube diameter.

It is worth noting that although the 3 mm condition exhibited the highest local peak turbulent kinetic energy at the breakup section, indicating the strongest local disturbance around the liquid column and the greatest refinement potential, the disturbance core along the centerline was slightly weaker than that under the 4 mm condition, and the high-disturbance region was distributed in a more localized manner. In addition, under actual production conditions, a small-diameter delivery tube is more likely to cause unstable melt delivery and clogging. By contrast, under the 4 mm condition, the local disturbance intensity at the breakup section remained at a relatively high level, while the centerline turbulent kinetic energy reached the maximum, and the high-turbulent-kinetic-energy region was distributed more continuously and uniformly. This indicates that the 4 mm condition achieved a more reasonable balance between liquid column refinement capability and flow stability.

**Figure 10:**
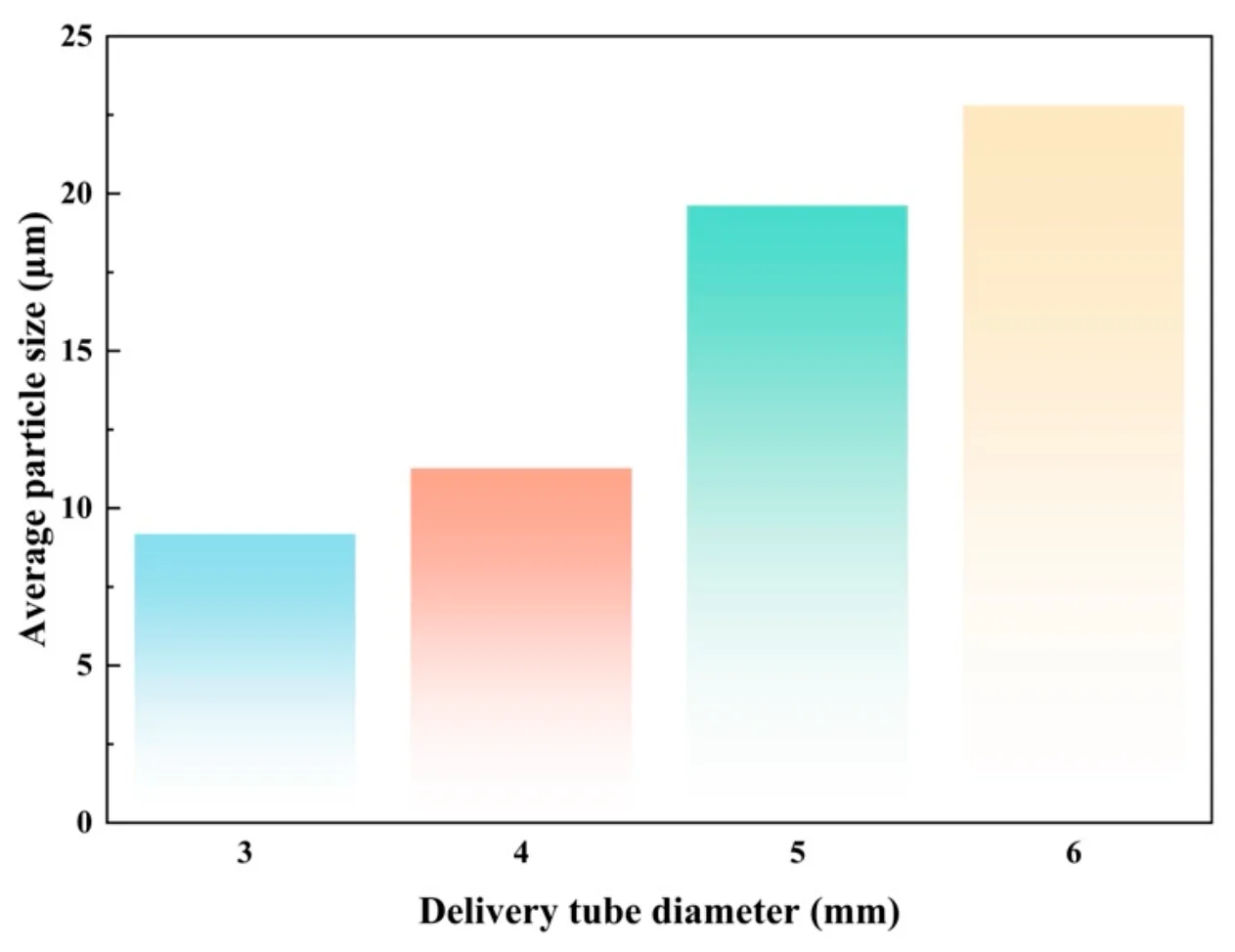
Comparison of the simulated average particle size under different delivery tube diameters.

The droplet size statistics obtained from the coupled VOF-DPM model are shown in Figure 10. As the delivery tube diameter increased from 3 to 4 mm, the average particle size increased from 9.176 to 11.265 μm, corresponding to an increase of only 22.77%, indicating that a moderate reduction in melt flow rate could improve the momentum matching between water and molten metal and thereby enhance shear-induced breakup. However, when the diameter was further increased to 5 and 6 mm, the average particle size increased markedly to 19.624 and 22.791 μm, respectively. In particular, under the 6 mm condition, the proportion of coarse particles increased significantly, and the average particle size exceeded that under the 4 mm condition by more than two times. The particle size distribution also shifted toward the coarse-size region, indicating a pronounced decline in the ability to generate fine powder.

Taken together, the velocity field, pressure field, and particle size statistics indicate that the delivery tube diameter systematically affects the primary breakup position, interfacial instability intensity, and secondary breakup efficiency by regulating the melt flow rate and the momentum ratio between water and molten metal. Mechanistically, the effect of delivery tube diameter is not limited to changing the melt flow rate; rather, it further influences the intensity of the interfacial shear layer, the development of the low-pressure entrainment zone, and the breakup location of the liquid column through its control of water-melt momentum matching. Under the 3 mm condition, the behavior may be characterized as “optimal refinement,” with the strongest liquid column instability and the finest droplets, but insufficient industrial stability. By contrast, the 4 mm condition may be described as “optimal overall balance,” as it maintains strong refinement capability while providing better melt delivery stability and a more favorable breakup process. In comparison, the 6 mm condition represents a typical “high-momentum penetration” regime, in which the liquid column is difficult to break up effectively, making it unfavorable for fine powder generation.

Therefore, based on the numerical simulation results, it can be preliminarily concluded that, within the range of conditions investigated in this study, the 4 mm delivery tube is more likely to be the optimal structural design because it provides a better balance between powder refinement capability and industrial operability. This conclusion is further validated by the subsequent industrial experiments.

## 4. Experimental Validation

To verify the reliability of the numerical simulation results and further evaluate the actual effect of delivery tube diameter on powder particle size characteristics and fine powder yield, comparative experiments were conducted using an industrial-scale water atomization system. During the experiments, key parameters including melting temperature, water pressure (120 MPa), nozzle structure, and spray angle were kept constant, while only the delivery tube diameter was varied (3, 4, 5, and 6 mm). The atomized powders were dried and sieved, followed by morphology observation and particle-size measurement, and the fine-powder yield was subsequently determined. The particle-size distributions were measured using a laser particle-size analyzer (Microtrac, Montgomeryville, PA, USA). The median particle size, D50, defined as the particle size corresponding to 50% of the cumulative particle-size distribution, was used as the representative experimental particle-size parameter.

**Figure 11:**
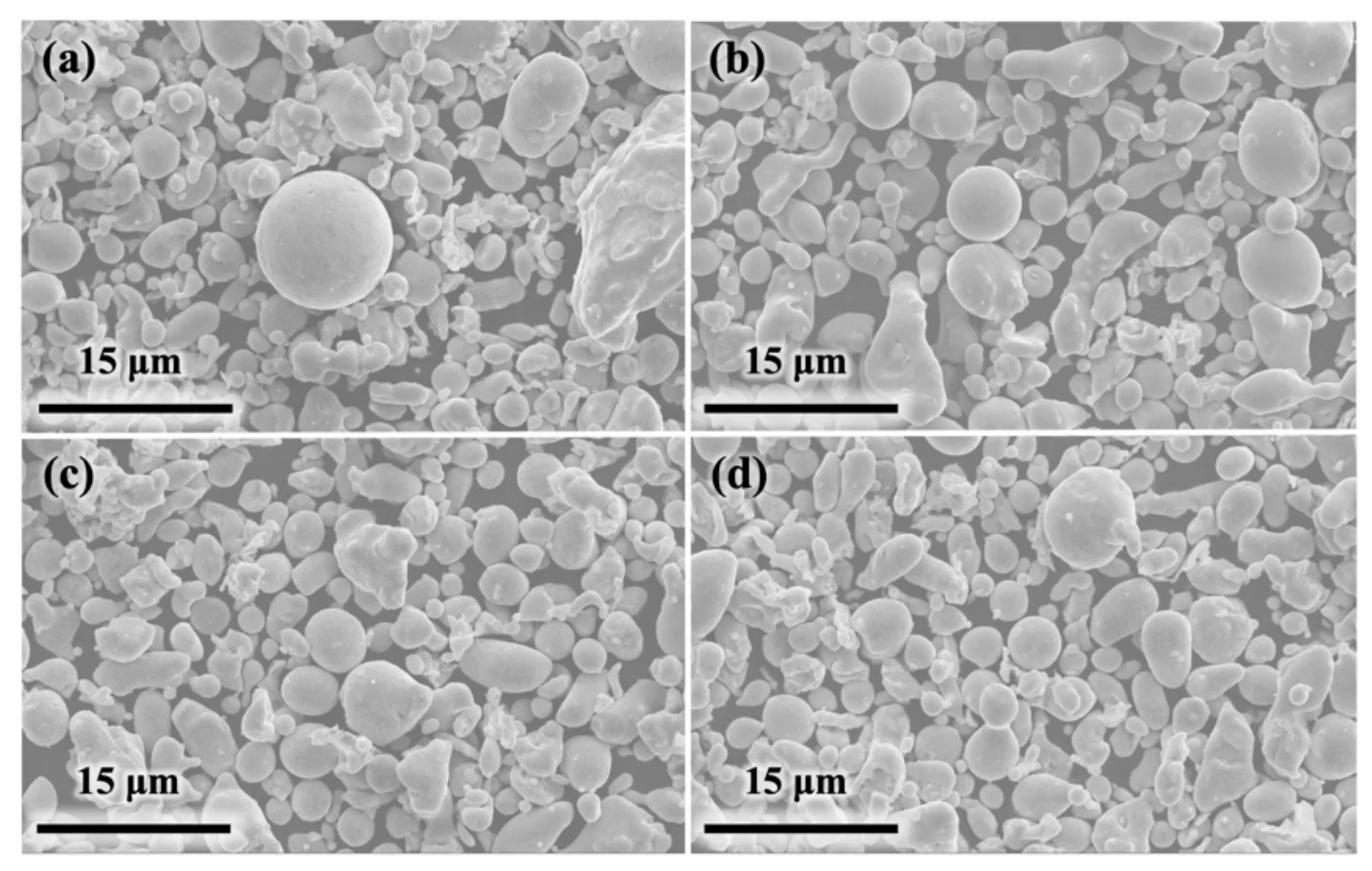
Representative SEM images of powders produced using delivery tubes with different diameters: (**a**) 3 mm; (**b**) 4 mm; (**c**) 5 mm; and (**d**) 6 mm. The images were obtained at the same magnification and are used primarily for qualitative comparison of particle morphology.

Figure 11 shows representative SEM images of the powders produced using delivery tubes with different diameters. Under all investigated conditions, the powders exhibited typical morphological characteristics of water-atomized powders, including near-spherical and irregular polyhedral particles, surface depressions, and satellite-particle adhesion. The overall morphologies under the different conditions partially overlapped, and the differences between individual SEM fields, particularly those for the 4 and 5 mm cases, were not pronounced. Therefore, these images are used mainly to provide a qualitative comparison of particle morphology, while the variation in particle size is evaluated primarily from the particle-size measurements and the fine-powder yields. Because each SEM image covers only a limited local field of view and includes partially overlapping particles, the existing images do not provide a sufficiently large and unbiased particle population for statistically reliable determination of the ratio between the maximum and minimum particle dimensions. Therefore, quantitative particle-shape analysis was not performed in the present study.

Under the 3 mm condition, particles with relatively large variations in size could be observed in the selected field of view. Under the 4 and 5 mm conditions, the powder particles showed broadly similar morphological features, and the differences between the two representative SEM images were limited. The 6 mm condition showed some relatively large and irregular particles, suggesting that incomplete breakup may occur more readily at the higher melt flow rate. Nevertheless, because each SEM image represents only a local field of view, these observations are regarded as qualitative evidence and are not used alone to determine the particle-size variation among the different conditions. The particle-coarsening tendency is assessed jointly using the particle-size distributions, simulated mean particle sizes, and experimental fine-powder yields.

**Figure 12:**
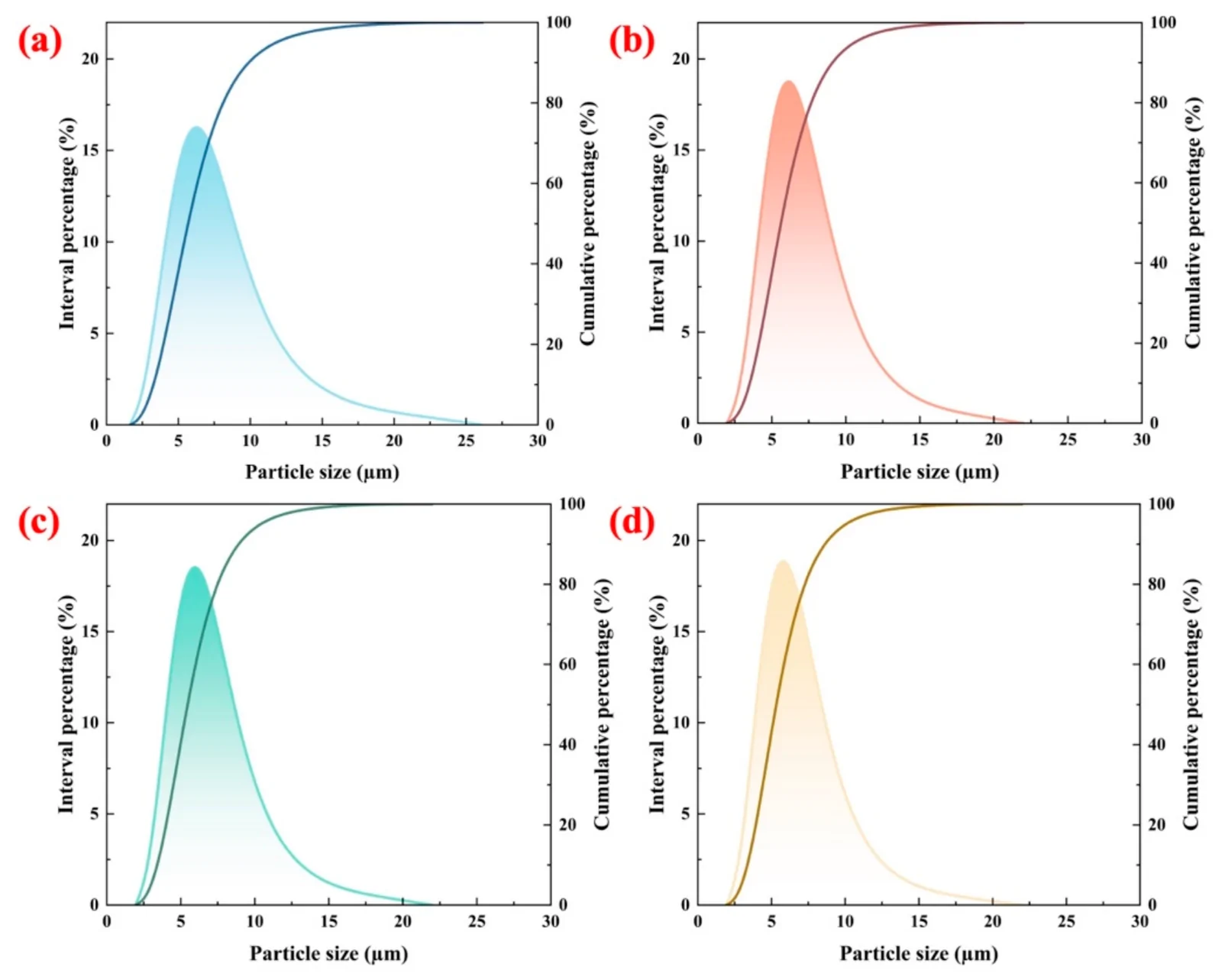
Particle-size distributions of powders produced using delivery tubes with different diameters: (**a**) 3 mm; (**b**) 4 mm; (**c**) 5 mm; and (**d**) 6 mm. The same particle-size range is used in all panels to facilitate comparison.

Figure 12 presents the particle-size distributions of the powders produced using delivery tubes with different diameters. The distribution curves under the four conditions partially overlap, particularly for the 4, 5, and 6 mm cases; therefore, the differences among the distributions are not readily distinguished from the local shapes of the curves alone. The 3 and 4 mm cases were mainly distributed in the relatively fine particle-size range. As the delivery-tube diameter increased to 5 and 6 mm, the distributions extended over a relatively wider range toward the coarse-particle side. Thus, rather than showing completely separated distribution curves, the experimental results indicate an overall tendency for the particle-size distribution to extend toward larger particle sizes with increasing delivery-tube diameter.

The overall variation in the experimental particle-size distributions is consistent with the trend of the simulated mean particle sizes. The simulated mean particle size increased only slightly when the delivery-tube diameter increased from 3 to 4 mm, but increased markedly when the diameter was further increased to 5 and 6 mm. In the experiments, the particle-size distributions for the 5 and 6 mm cases extended further toward the coarse-particle range, while the corresponding fine-powder yields decreased. These consistent trends support the analysis that the momentum matching between the water jets and the molten metal affects the breakup and refinement efficiency.

It should be noted that the particle-size distribution curves partially overlap. Therefore, the particle-coarsening tendency discussed here refers to an overall trend evaluated jointly from the experimental particle-size distributions, simulated mean particle sizes, and fine-powder yields, rather than from the visual separation of the distribution curves alone.

To facilitate a more direct quantitative comparison between the simulation and experiment, the experimentally measured mean particle sizes were further extracted from the original laser particle-size analysis results. For delivery-tube diameters of 3, 4, 5, and 6 mm, the experimental mean particle sizes were 9.0, 10.3, 15.1, and 19.6 μm, respectively. The corresponding simulated mean particle sizes were 9.176, 11.265, 19.624, and 22.791 μm, respectively. Although the absolute values obtained from the simulation and experiment were not identical, both results showed the same overall variation trend: the particle size changed only slightly from the 3 mm condition to the 4 mm condition, but increased more evidently when the delivery-tube diameter was further increased to 5 and 6 mm. This agreement indicates that the coupled VOF-DPM model reasonably captures the influence of delivery-tube diameter on particle-size evolution. The simulated and experimental mean particle sizes are summarized in Table 6 for a direct comparison.

**Figure 13:**
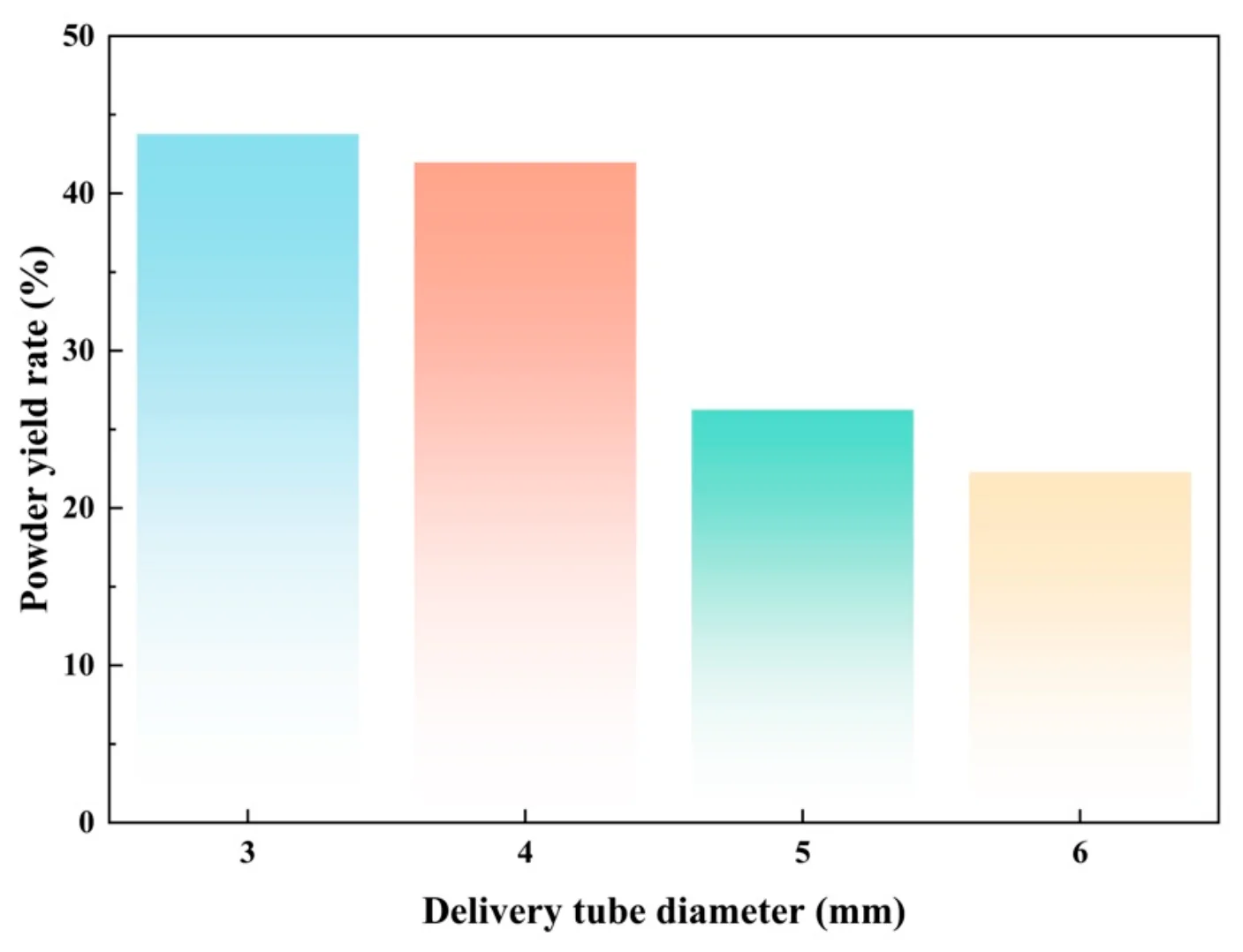
Fine powder yield obtained in the experiments using delivery tubes with different diameters.

**Figure 14:**
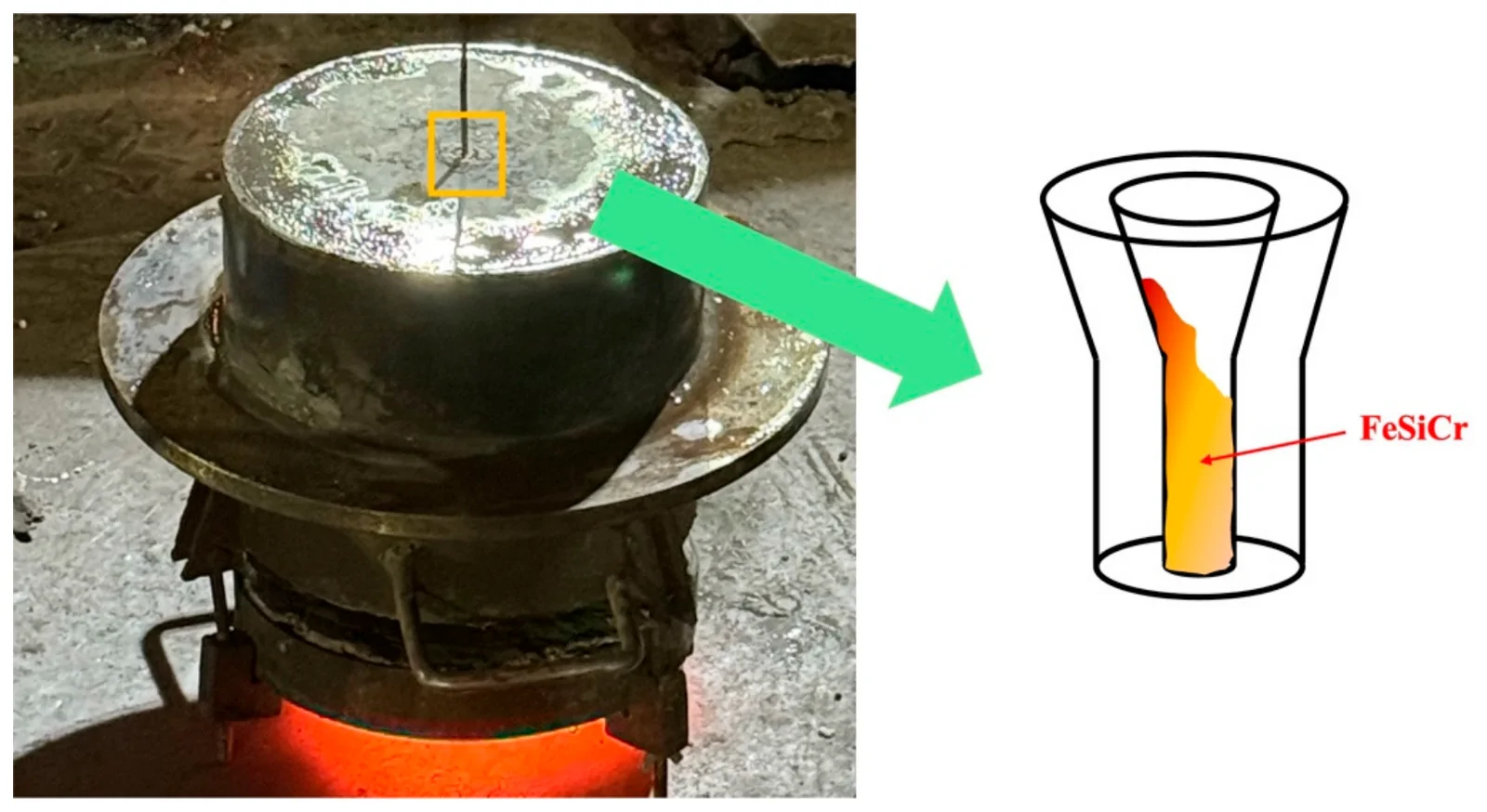
Schematic illustration of delivery tube clogging. The yellow box indicates the clogging region observed during actual production, and the green arrow points to the corresponding schematic representation of FeSiCr accumulation inside the delivery tube.

In addition to the comparison of the simulated and experimental mean particle sizes, the fine-powder yield under different delivery-tube diameter conditions was evaluated to further quantify the atomization performance, as shown in Figure 13. Here, the fine powder yield was defined as the proportion of powder with a particle size of 5–6 μm in the final product. The 3 mm delivery tube produced the highest fine powder yield, reaching 43.77%, which was only 4.3% higher than that under the 4 mm condition (41.96%). However, the 3 mm condition was more prone to clogging during actual production, which had a substantial adverse effect on continuous operation, as illustrated in Figure 14. By contrast, the fine powder yields under the 5 and 6 mm conditions decreased significantly to only 26.25% and 22.28%, respectively, with the 6 mm condition showing the most pronounced decline, representing a 46.9% reduction compared with the 4 mm condition. This trend is consistent with the particle size distribution results, indicating that an excessively high melt flow rate weakens the shearing and tearing effects of the water jets on the molten metal, thereby reducing the efficiency of fine powder generation.

Based on the SEM observations, particle-size distribution analysis, comparison of the simulated and experimental mean particle sizes, and fine-powder yield results, the 4 mm delivery tube provided a favorable balance between particle refinement and production stability under the processing conditions investigated in this study. The experimental mean particle sizes exhibited the same overall variation trend as the simulated values, with only a limited increase from 3 to 4 mm and a more evident increase at 5 and 6 mm. Together with the fine-powder yield results, this agreement supports the capability of the coupled VOF-DPM model to predict the influence of delivery-tube diameter on breakup behavior and particle-size evolution during water atomization.

## 5. Conclusions

This study established a coupled VOF-DPM numerical model to investigate the effect of delivery tube diameter on the flow and breakup behavior during water atomization of FeSiCr alloy, and the model was systematically validated through industrial experiments. The main conclusions are as follows:

(1) The delivery tube diameter is a key structural parameter affecting the melt flow rate, initial momentum flux, and the momentum matching relationship between water and molten metal during the water atomization of FeSiCr. As the delivery tube diameter increased from 3 to 6 mm, the primary breakup position of the liquid column shifted downstream from 94.6 to 236.5 mm, while the peak negative pressure along the centerline decreased from −41.12 to −31.65 kPa, indicating enhanced melt penetration capability and weakened breakup-promoting effects of the water flow in the near-field region.
(2) The velocity field, pressure field, and turbulent kinetic energy results indicate that an appropriate melt delivery condition is favorable for forming a stronger interfacial shear layer, a more pronounced low-pressure entrainment zone, and a more effective core disturbance region, thereby promoting liquid column instability, liquid film tearing, and droplet refinement. Under the 3 mm condition, the simulated average particle size was the smallest, at 9.176 μm. When the delivery tube diameter increased to 5 and 6 mm, the average particle size increased to 19.624 and 22.791 μm, respectively, indicating that breakup was significantly delayed under large-diameter conditions and that the fine powder generation capability decreased markedly.
(3) The experimentally measured mean particle sizes of 9.0, 10.3, 15.1, and 19.6 μm for the 3, 4, 5, and 6 mm delivery tubes, respectively, showed the same overall variation trend as the simulated mean particle sizes. Although the 3 mm delivery tube produced the finest powder and the highest fine-powder yield, it also posed risks of clogging and unstable melt delivery during continuous production. By contrast, the 4 mm delivery tube achieved a better balance among refinement capability, particle-size uniformity, and operational stability and is therefore a more suitable structural design for industrial application under the conditions investigated in this study.

## Figures and Tables

**Table 1:** Comparison of molten steel flow rate variation with melt delivery tube diameter.

Delivery Tube Diameter (mm)	Metal Liquid Flow (kg/s)
3	0.1
4	0.2
5	0.263
6	0.368

**Table 2:** Dimensions of the delivery tube.

Geometric Parameter	Length (mm)
a	3
b	13
c	10
d	15
e	3, 4, 5, 6

**Table 3:** Summary of governing equations.

The Governing Equations	Nomenclature
1. Mass conservation $\frac{\partial \rho }{\partial t}+\nabla \cdot (\rho \vec{v})=0$ (1)	ρ is the density t is the time $\vec{v}$ is the velocity vector
2. Momentum conservation $\frac{\partial }{\partial t}(\rho \vec{v})+\nabla \cdot (\rho \vec{v}\times \vec{v})=-\nabla p+\nabla \cdot (\bar{\bar{\tau }})+\rho \vec{g}+\vec{F}$ (2) $\bar{\bar{\tau }}=\mu [(\nabla \vec{v}+\nabla {\vec{v}}^{T})-\frac{2}{3}\nabla \cdot \vec{v}I]$ (3)	$\bar{\bar{\tau }}$ is the stress tensor $\vec{g}$ is the gravitational acceleration $\vec{F}$ is the external volume force I is the unit tensor
3. Energy conservation $\frac{\partial }{\partial_{t}}\left(\rho E\right)+\nabla \cdot \left(\vec{v}\left(\rho E+P\right)\right)=\nabla \cdot \left(k_{eff}\nabla T\right)+S_{T}$ (4) $S_{T}=\left(\overset{=}{\tau }\cdot \nabla \right)\vec{v}$ (5)	E is the internal energy $k_{eff}$ is the effective conductivity $S_{T}$ is the viscous dissipation term
4. VOF $\begin{array}{l} \frac{\alpha_{q}^{n+1}\rho_{q}^{n+1}-\alpha_{q}^{n}\rho_{q}^{n}}{\Delta t}V+\sum_{f}(\rho_{q}^{n+1}U_{f}^{n+1}\alpha_{q,f}^{n+1})=\\ {}[S_{\alpha_{q}}+\sum_{p=1}^{n}({\overset{\cdot }{m}}_{pq}-{\overset{\cdot }{m}}_{qp})]V \end{array}$ (6)	n+1 is the present time step n is the previous time step $\alpha_{q}^{n+1}$ is the volume fraction value of the mesh cell at time step n + 1 $\alpha_{q}^{n}$ is the volume fraction value of the mesh cell at time step n $\alpha_{q,f}^{n+1}$ is the interfacial value of the volume fraction for the q-th phase at time step n + 1 $U_{f}^{n+1}$ is the volumetric flow rate across the face at time step n + 1 V is the cell volume
5. DPM $m_{p}\frac{d{\vec{u}}_{p}}{dt}=m_{p}\frac{\vec{u}-{\vec{u}}_{p}}{\tau_{r}}+m_{p}\frac{\vec{g}(\rho_{p}-\rho )}{\rho_{p}}+\vec{F}$ (7) τr=ρpdp218μ24CdRe (8) Re≡ρdpu→p−u→μ (9)	$m_{p}$ is the particle mass $\vec{u}$ is the fluid phase velocity ${\vec{u}}_{p}$ is the particle velocity ρ is the fluid density $\rho_{p}$ is the density of the particle $\vec{F}$ is an additional force $\tau_{r}$ the droplet or particle relaxation time μ is the molecular viscosity of the fluid $d_{p}$ is the particle diameter Re is the relative Reynolds number

**Table 4:** Thermophysical properties of the materials used in the numerical model.

	Air	Water-Liquid	FeSiCr
Density (kg·m^−3^)	1.225	998.2	7080
Specific Heat Capacity (J·kg^−1^·K^−1^)	1006.43	4182	710
Thermal Conductivity (W·m^−1^·K^−1^)	0.0242	0.6	25.46
Viscosity (kg·m^−1^·s^−1^)	1.7894 × 10^−5^	1.003 × 10^−3^	5.76 × 10^−3^

**Table 5:** Primary breakup positions measured downward from the bottom of the tundish for different delivery-tube inner diameters.

Delivery Tube Diameter (mm)	Primary Breakup Position (mm)
3	94.6
4	138.6
5	185.9
6	236.5

**Table 6:** Comparison of the simulated and experimental mean particle sizes under different delivery-tube diameter conditions.

Delivery-Tube Diameter (mm)	Simulated Mean Particle Size (μm)	Experimental Mean Particle Size (μm)
3	9.176	9.0
4	11.265	10.3
5	19.624	15.1
6	22.791	19.6

## Data Availability

The original contributions presented in this study are included in the article. Further inquiries can be directed to the corresponding authors.
